# Light‐Driven PAA Adhesive: A Green Bonding Platform Integrating High‐Performance, Environmental Resilience, and Closed‐Loop Recyclability

**DOI:** 10.1002/advs.202503788

**Published:** 2025-04-25

**Authors:** Xueying Fu, Jingtian Chen, Yuqi Zhao, Yanan Liu, Chenyang Xie, Xuhang Zhang, Yingdan Liu, Jingyue Yang

**Affiliations:** ^1^ State Key Laboratory of Metastable Materials Science and Technology Nano‐biotechnology Key Lab of Hebei Province Applying Chemistry Key Lab of Hebei Province Yanshan University Qinhuangdao 066004 China; ^2^ Center for Advanced Structural Materials State Key Lab of Metastable Materials Science and Technology College of Materials Science and Engineering Yanshan University Qinhuangdao 066004 China

**Keywords:** green, recyclable, super‐strong adhesive, ultra‐stable, visible light catalysis

## Abstract

The increasing demand for environmentally benign materials has driven significant interest in water‐based adhesives due to their low toxicity and ecological advantages. However, conventional formulations face persistent challenges including limited bonding strength, complex manufacturing processes, and compromised storage stability. To address these limitations, a polyacrylic acid‐based aqueous adhesive (PAA) is developed through a novel visible‐light catalytic platform. This approach ensures a mild catalytic cycle, thereby promoting sustained stability. The strategic integration of hydrogen bonding, electrostatic interactions, and mechanical interlocking enhances interfacial adhesion. Notably, the adhesive demonstrates an adhesion strength of up to 20.86 MPa on wood and 12.91 MPa on bamboo substrates. Its composition confers stability across diverse environmental conditions, including extreme temperature variations (−196 °C–200 °C), prolonged storage (> 270 days), and resistance to mechanical stress and solvent exposure. Furthermore, PAA exhibits full recyclability through a water‐mediated dissociation and recovery process. This study represents a pioneering application of novel visible‐light catalysis in adhesive synthesis, advancing the development of sustainable high‐performance bonding systems.

## Graphical abstract

This investigation introduces the formulation of a cutting‐edge, robust polyacrylic aqueous adhesive (PAA), synthesized through the employment of visible‐light initiated polymerization. The adhesive demonstrates minimal energy demand, environmental friendliness, robust adhesive properties (20.86 MPa on wood), remarkable thermal stability (with operational capability from −196 °C to 200 °C), enhanced durability (over 270 days), and resistance to mechanical shock and organic solvents. Furthermore, the adhesive boasts recyclability, underscoring its potential for sustainable applications.

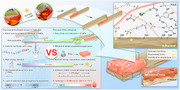

## 1 Introduction

The extensive use of modern adhesives in automobiles,^[^ ^1^ ^]^ electronics,^[^ ^2^ ^]^ furniture,^[^ ^3^ ^]^ biomedicine,^[^ ^4^, ^5^, ^6^, ^7^, ^8^, ^9^ ^]^ footwear,^[^ ^10^ ^]^ packaging,^[^ ^11^, ^12^ ^]^ and transportation^[^ ^13^ ^]^ highlights their essential role in everyday applications while simultaneously raising environmental awareness.^[^ ^14^ ^]^ While conventional adhesives demonstrate cost‐effective performance, they incur substantial ecological costs through formaldehyde emissions in wood composites and persistent volatile organic compound (VOC) release during service.^[^ ^15^, ^16^ ^]^ End‐of‐life management challenges of permanent adhesives and waste adhesive processing further environmental burdens.^[^ ^17^, ^18^, ^19^, ^20^ ^]^ In response to these challenges, many researchers have shifted toward non‐volatile solvent‐based adhesives^[^ ^21^, ^22^ ^]^ and water‐based adhesives as viable alternatives to traditional solvent‐containing options. Recently, water‐based adhesives such as polyvinyl alcohol and acrylic adhesives have gained attention due to their environmentally friendly and non‐toxic characteristics.^[^ ^23^, ^24^ ^]^ Nevertheless, despite these environmental benefits, water‐based adhesives usually suffer from diminished adhesive strength (below 3 MPa).^[^ ^25^, ^26^ ^]^ Furthermore, their production is often protracted and labor‐intensive, involving complex synthesis processes,^[^ ^27^, ^28^ ^]^ which consequently leads to substantial energy consumption.

In the context of sustainable development, solar energy as a clean and renewable resource can effectively reduce dependence on fossil fuels, thereby substantially reducing long‐term energy expenditures. While ruthenium‐based photocatalysis (e.g., Ru(II)/S₂O₈^2^⁻ systems) has demonstrated exceptional kinetic advantages in visible‐light‐activated platforms for applications spanning printable adhesives to regenerative biomaterials,^[^ ^29^, ^30^, ^31^, ^32^ ^]^ critical limitations persist in conventional formulations. Current systems inherently rely on potent oxidizing agents—specifically nitrobenzenediazonium salt (NBD) or ammonium persulfate (APS)—which, while facilitating photopolymerization, concurrently induce spontaneous thermal decomposition under ambient conditions within a week irrespective of photonic activation, fundamentally restricting their storage stability and industrial scalability.^[^ ^30^ ^]^ Furthermore, the adhesion strength of these systems remains confined to the kilopascal range (KPa), necessitating substantial improvement to meet rigorous performance criteria. To address these dual challenges, we present a mild photocatalytic system engineered to mitigate degradation through optimized electron transfer pathways, coupled with enhanced interfacial adhesion achieved via strategic incorporation of hydrogen‐bonding motifs.

**Figure 1:**
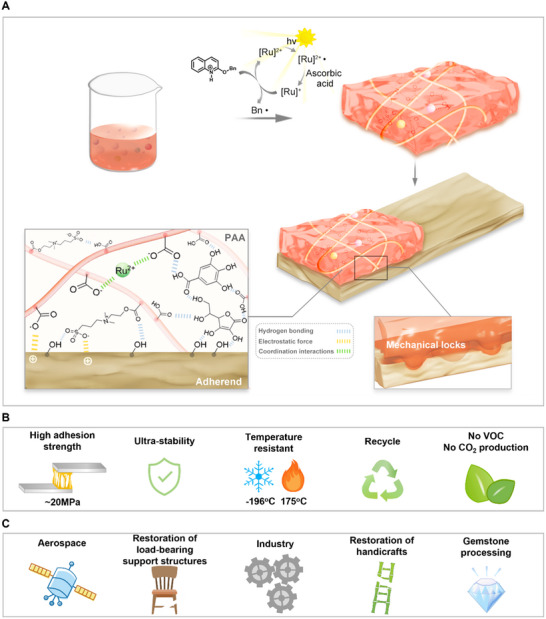
Preparation and properties of the PAA adhesive. A) Preparation and adhesion force of the PAA adhesive. B) Advantages of the PAA adhesive. C) Applications of the PAA adhesive.

Herein, we report a solar‐driven, water‐based, recyclable adhesive that exhibits superior adhesion strength across a range of substrates, coupled with exceptional stability (**Figure** **1**). This study employs non‐toxic ruthenium (Ru II) as a photocatalyst to facilitate the generation of benzyl radicals from 2‐benzyloxyquinoline under visible light, thereby initiating the polymerization of acrylic monomers. This gentle catalytic process guarantees the adhesive's enduring stability. In this system, Ru(bpy)_3_^2+^ functions both as a photocatalyst and as a coordination site for bonding. Ascorbic acid not only serves as a crucial reductant in the visible‐light photocatalytic cycle but also contributes additional hydrogen bonding. Moreover, the electrostatic interactions provided by 2‐(N‐3‐Sulfopropyl‐N, N‐dimethyl ammonium) ethyl methacrylate (DMAPS) and hydrogen bonding from gallic acid (GA) and carboxylic groups bolster both internal and interfacial adhesion. These non‐covalent interactions are instrumental in endowing the novel adhesive system with its robust and reversible properties. The raw materials amalgamate to form a transparent solution, which undergoes a spontaneous transformation into a hydrogel adhesive upon illumination, obviating the requirement for stirring. Subsequent application to diverse substrates is facilitated. Dehydration leads to the formation of a rigid lock between the contracting surface and the coacervates. This novel PAA adhesive demonstrates exceptional adhesion strength on rough surfaces (20.86 MPa on wood) and hydrophilic smooth surfaces (11.03 MPa on stainless steel). Both the pre‐polymer solution and the hydrogel post‐light exposure exhibit outstanding stability, with no degradation detected upon storage for extended periods. The adhesion strength of substrates bonded with PAA remained consistent after 270 days, the longest duration tested to date, thus setting no definitive upper limit for its stability retention. Furthermore, this innovative PAA adhesive possesses the ability to withstand a broad temperature spectrum from −196 °C—200 °C, making it suitable for aerospace and automotive applications. Its detachment using water allows for the recycling of products and materials, while the adhesive itself can be reshaped and reused, retaining its adhesion strength across multiple cycles. When compared to existing water‐based adhesives, this adhesive not only substantially elevates adhesion strength and long‐term stability but also achieves near‐zero energy consumption during both preparation and application.

## 2 Results

### 2.1 Preparation and Characterization

**Figure 2:**
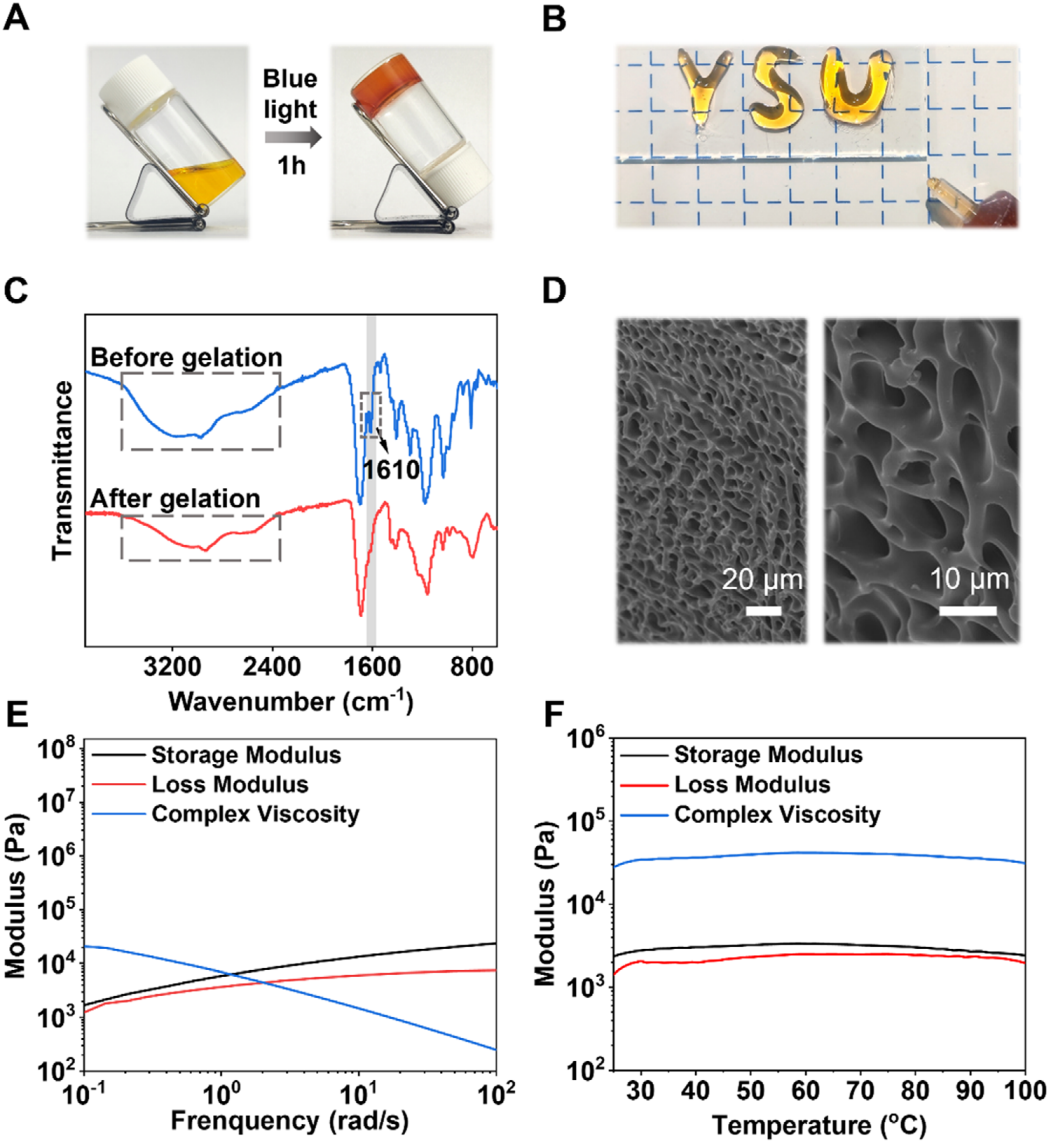
Preparation and characterization of the PAA adhesive. A) Optical image of the gelation process of the PAA adhesive subsequent to exposure to blue light irradiation. B) Optical image of the injectable hydrogel adhesive prior to curing. C) FT‐IR spectra of the PAA adhesive before and after gelation. D) SEM images of the lyophilized PAA adhesives. E) Rheological characterization of the PAA adhesive at ambient temperature conditions. F) Temperature‐dependent rheological evaluation of the PAA adhesive.

During the preparation of the PAA adhesive, polymerization is initiated by trace amounts of benzyl radicals emanating from minute concentrations of 2‐benzyloxyquinoline (Figures S1 and S2, Supporting Information), as described in the literature.^[^ ^33^ ^]^ An insufficient concentration of acrylic acid leads to inadequate adhesion strength, whereas an excessively elevated concentration raises the viscosity of the hydrogel, thereby complicating the coating process and ultimately diminishing the resultant adhesion. An optimal acrylic acid concentration of 32% (w/w) has been established (Figure S3, Supporting Information). Correspondingly, 0.02% (w/w) of the photocatalyst Ru(bpy)_3_^2+^ is deemed sufficient to achieve maximal adhesion effectiveness. Moreover, the incorporation of small amounts of GA and DMAPS contributes to the enhancement of overall adhesion by providing additional binding sites. The comprehensive adhesive formulation and optimization data are presented in Table S1 (Supporting Information). The preparation process of our PAA adhesive is notably simple and efficient. Upon amalgamation of the constituent components, a transparent yellow solution is obtained, which promptly converts into an injectable gel upon exposure to blue light (**Figure** **2**A,B). The fluid nature of this gel augments the contact area between the adhesive and the substrate, thereby ensuring optimal interfacial adhesion strength. Notably, this entire process is executed without the need for supplementary heating or stirring, obviating energy consumption and consequently lowering production costs, while adhering to principles of sustainable development.

The accomplishment of the polymerization reaction, thereby substantiating the effective preparation of the PAA adhesive, was validated through Fourier‐transform infrared spectroscopy (FT‐IR) and scanning electron microscopy (SEM). A comparative analysis of the infrared spectra of the PAA adhesive before and subsequent to gelation exhibited the disappearance of the C ═ C stretching vibration peak at 1610 cm⁻¹ in the pre‐polymer solution upon illumination (Figure 2C). This observation signifies a substantial decrease in the content of double bonds, strongly suggesting that the acrylic acid monomers have successfully undergone polymerization. Furthermore, polymerization brings the carboxyl groups into closer proximity, thereby facilitating the establishment of hydrogen bonds and coordination interactions, which in turn reduces their degrees of freedom. This observation is consistent with the observed reduction in the intensities of the C ═ O and ─OH stretching vibration peaks, located at 1700 cm^−1^ and 3100 cm⁻¹, respectively. Additionally, the SEM images of the lyophilized PAA gel reveal a dense and uniform porous network structure, further validating the successful completion of the polymerization reaction (Figure 2D). The rheological behavior of the PAA adhesive is illustrated in Figure 2E,F. Within the frequency range of 0.1–100 rad s^−1^, both the storage modulus and loss modulus increase with frequency. The comparable magnitudes of these moduli suggest that the PAA adhesive behaves as a gel, which aligns with its physical state and facilitates the coating process, thereby enhancing its user‐friendliness. Moreover, the viscosity of the PAA adhesive maintains relatively constant across a range of temperatures, indicating its good thermal stability and potential applicability over a broad temperature spectrum.

### 2.2 Adhesion Properties

**Figure 3:**
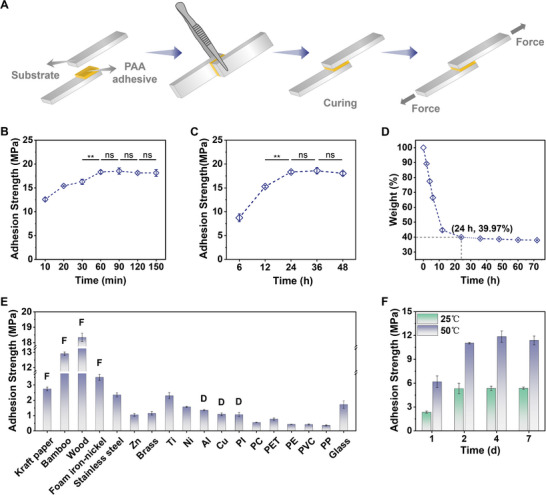
Adhesion performance. A) Scheme illustration of the adhesive application process. B) Adhesion strengths under varying exposure durations to light (on wood). C) Adhesion strengths across diverse curing time intervals. D) Dehydration process of the PAA adhesive under ambient conditions. E) Adhesion strengths of the PAA adhesive on various substrate surfaces (with “F” indicating Substrate Fracture and “D” indicating Substrate Deformation, curing condition: 24 h at 25 °C). F) Adhesion strengths under different curing conditions on stainless steel. Data were presented as means ± standard deviation (*n* = 3). Significant levels were indicated as ns = no significant difference (*p* > 0.05), ^**^ *p* < 0.01.

In addition to its expeditious and energetically efficient fabrication protocol, the application and curing of the PAA adhesive demonstrate a comparable level of convenience. Following application between two substrates, the adhesive requires only a minimal application of pressure, achievable through the use of forceps or equivalent tools, to initiate the curing process to occur at ambient temperatures (**Figure** **3**A). The lap shear test stands as a robust and extensively employed methodology for assessing adhesive performance, providing a substantial amount of reproducible data. The adhesive strength is quantified as the maximum force per unit area of the adhesion at the point of failure. Experimental investigations into adhesive strength under various illumination durations have revealed that a substantial adhesion value of 12.59 MPa is achieved within a mere 10 min of light exposure. Notably, the adhesive strength increases progressively with extended light exposure, culminating in a peak of 18.33 MPa after prolonged illumination (Figure 3B). Upon one hour of illumination, the adhesive strength reaches its maximum and stabilizes, suggesting that the chemical reactions within the prepolymer solution are essentially completed at this stage.

Further investigations into curing times were conducted on wood substrates, with coated samples subjected to room‐temperature curing for durations of 6, 12, 24, 36, or 48 h (Figure 3C). The results indicated that a curing interval of 24 h yielded the highest bond strength of 18.33 MPa, followed by a 12‐h curing duration with a nearly equivalent bond strength of 15.28 MPa. The curing duration was found to be closely associated with the evaporation rate of moisture from the adhesive hydrogel (Figure 3D). The weight loss of the PAA hydrogel under ambient conditions exhibited a robust correlation with the curing outcomes. After 24 h of drying, the adhesive's weight reduction was essentially complete, decreasing to 39.97% of its initial mass, which coincided with the cured material ratio of 37.29%, suggesting that the curing process was nearly finished. The weight loss observed at 12 h was also closely approximated to that at 24 h, indicating that the curing process was approaching completion at this stage.

The adhesive bonding mechanism encompasses a complex interplay of processes, including intermolecular bonding, electrostatic bonding, and mechanical interlocking, among others. In this PAA adhesive system, besides the entanglement of the polymer's extended chains, the key interactions within the adhesive and at the adhesive‐substrate interface are predominantly mediated by hydrogen bonding and electrostatic forces. As depicted in Figure 3E, the adhesive demonstrated significantly higher average adhesion strengths on rough substrates such as wood, bamboo, Kraft paper, and foam iron‐nickel, as compared to smooth substrates like copper (Cu), aluminum (Al), titanium (Ti), stainless steel, glass, polyimide (PI), and polyethylene terephthalate (PET). Notably, the adhesive strength on wood and bamboo reached 18.33 and 12.91 MPa, respectively, with substrate failure occurring in both cases. To the best of our knowledge, these values represent the highest reported adhesion strengths in the literature to date for these substrates. Similarly, the adhesion strength for Kraft paper and foam iron‐nickel was ≈3 MPa, with substrate failure also observed. The correlation between substrate surface roughness and adhesion strength suggests the presence of mechanical interlocking at the rough interfaces. SEM images of the adhesive‐wood interface further validated the occurrence of mechanical interlocking at the interface (Figure S4, Supporting Information).

Moreover, the adhesion strength displayed on hydrophilic substrates, such as stainless steel and Ti (2.35/2.30 MPa), consistently outperforms that on hydrophobic substrates, such as PI (1.06 MPa), a trend that aligns with the expected behavior of water‐based adhesives. Additionally, it is recognized that for dense, smooth substrates akin to those mentioned, the observed diminished adhesion strength can be attributed to a considerable reduction in the rate of moisture evaporation. This suggests that the adhesive's curing efficacy may not achieve its optimal potential under ambient conditions within 24 h. Consequently, stainless steel and titanium were selected for the re‐evaluation and optimization of curing parameters. By extending the curing time at ambient temperature and at 50 °C, it was ascertained that both substrates require prolonged curing periods to achieve optimal adhesive properties. Upon elevating the curing temperature to 50 °C and extending the curing duration to 2 and 4 days, the adhesion strengths on stainless steel and titanium substrates were found to reach 11.03 and 6.08 MPa, respectively (Figure 3F; Figure S5, Supporting Information).

Thermogravimetric analysis (TGA) of the PAA adhesives under various curing conditions offers validation for the degree of curing achieved (Figure S6, Supporting Information). The comparison of the TGA curves for the uncured PAA gel, the adhesive cured for 24 h at room temperature, and the adhesive cured for 48 h at 50 °C reveals a progressive decrease in mass reduction with increasing temperature from room temperature to 148 °C, corresponding to 60.0%, 9.0%, and 3.9% of the initial mass, respectively. The uncured PAA adhesive exhibits a rapid loss of moisture during this process, resulting in a residual mass that approaches the net mass of the adhesive excluding water (37.29%). In contrast, the thermogravimetric curves of the cured materials do not exhibit such a precipitous decline, indicating the evaporation of most of the moisture within the materials following curing. Notably, the TGA curve corresponding to the adhesive cured for 48 h at 50 °C demonstrates the least degree of mass loss, suggesting a more comprehensive curing process.

### 2.3 Robust Durability and Stability

**Figure 4:**
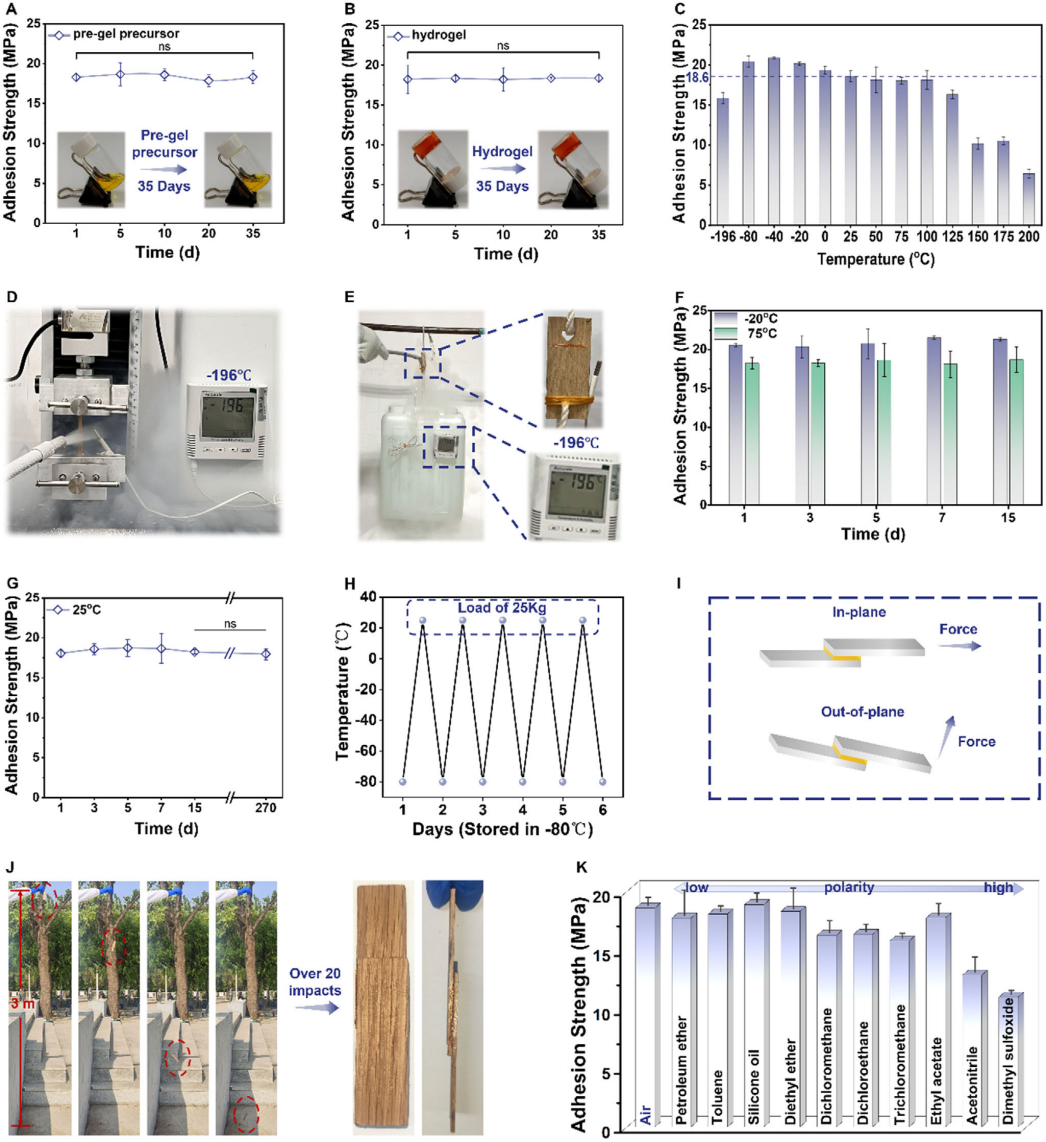
Durability and Stability of the PAA adhesive. A) Adhesion strengths of the PAA adhesive after long‐term storage of the pre‐gel precursor (on wood). B) Adhesion strengths of the PAA adhesive after long‐term storage of the hydrogel (on wood). C) Adhesion strengths of the PAA adhesive at various temperatures (on wood). D) Lap shear test of the PAA adhesive at −196 °C with a liquid nitrogen spray gun. E) Load test of the PAA adhesive at −196 °C with a liquid nitrogen spray gun. F) Adhesion strengths of the PAA adhesive at −20 °C and 75 °C over different durations (on wood). G) Adhesion strengths of the PAA adhesive at ambient temperature over different durations (on wood). H) Load test of the PAA adhesive with alternating temperature cycles from −80 °C and room temperature. I) Schematic diagram of in‐plane and out‐of‐plane forces. J) Drop test from a height of 3 meters with a bonded area of 10 cm^2^. K) Adhesive strengths of the PAA adhesive in different organic solvents (on wood). Data were presented as means ± standard deviation (*n* = 3). Significant levels were indicated as ns = no significant difference (*p* > 0.05).

The stability of the adhesive is a critical criterion for ensuring its integrity throughout the storage and transportation processes. Furthermore, the adhesive's resilience in diverse environmental conditions, particularly within the confines of extreme environments, is pivotal for the enduring preservation and operational efficacy of bonded components. In light of these considerations, a comprehensive investigation was conducted to evaluate the storage stability, thermal stability, and solvent resistance of the PAA adhesive. Both the pre‐gel precursor and the hydrogel formed subsequent to irradiation displayed no discernible changes in appearance or adhesive performance following prolonged storage at room temperature for over a month (**Figure** **4**A,B). This observation underscores their remarkable stability and effectively counters the prevalent issue of expedited adhesive degradation that is often encountered in photocatalytic adhesives during storage periods.^[^ ^30^ ^]^

In scenarios of specific extreme environmental conditions, adhesives must withstand significant temperature excursions. For instance, in high‐latitude regions, winter temperatures can plummet to as low as −40 °C and −30 °C, while summer temperatures can rise above 20 °C. In environments of even greater extremity, such as those encountered in spacecraft, temperature variations between the dark and sunlit sides can span from −100 °C and 100 °C.^[^ ^34^ ^]^ To address these challenges, lap shear tests were conducted on bonded wood samples subjected to various temperature conditions for 12 h, including −80 °C, −40 °C, −20 °C, 0 °C, ambient temperature, 50 °C, 75 °C, 100 °C, 125 °C, 150 °C, 175 °C, and 200 °C (Figure 4C). Within the tested temperature range of −80 °C–100 °C, the adhesion strength remained largely unaffected. The minor increase in strength observed at lower temperatures may be attributed to the enhanced hardness of wood under such conditions. However, as the temperature exceeded 125 °C, the adhesive's color darkened, and there was a decline in adhesion strength, although substrate failure was the cause of breakage in all cases. To evaluate the adhesive's performance at extremely low temperatures, fully cured wood samples were subjected to a liquid nitrogen spray, achieving a temperature of −196 °C (Figure 4D). Despite this extreme cooling, the adhesive retained an exceptional adhesion strength of 15.84 MPa. Moreover, to assess the practical viability of the adhesive at cryogenic temperatures, low‐temperature load tests were conducted. In these tests, a 25 kg water bucket was suspended from bonded wood pieces with an adhesive area of ≈12 cm^2^ and maintained at −196 °C using liquid nitrogen spray (Movie S1, Supporting Information). As depicted in Figure 4E, the bucket remained securely attached without any detachment or movement during the test. Subsequently, the bonded wooden samples were subjected to alternating cycles between −80 °C and room temperature, followed by load testing at room temperature (Figure 4H). After more than five cycles, the samples continued to bear a 25 kg load without any signs of separation or displacement. Furthermore, bonded wood samples were stored at low temperatures (−25 °C), room temperature, and high temperature (75 °C), with lap shear tests conducted after 1, 3, 5, 7, and 15 days (Figure 4F,G). The results confirmed the consistency of the adhesive performance. Notably, the sample was stored at room temperature for up to 270 days, with no change in adhesion properties observed. Even samples stored for over a month under these three temperature conditions (with an adhesive area of ≈12 cm^2^) still supported a 25 kg load without any fractures or displacement (Figure S7, Supporting Information). These findings highlight the exceptional stability of this novel adhesive across a broad spectrum of temperatures and temperature fluctuations. Beyond environmental temperature, the PAA adhesive demonstrated robust tolerance to humidity variations. At humidity levels below 60%, the adhesion strength remained above 16 MPa; however, a marked decrease in adhesion strength was observed only when humidity reached 80% (Figure S8, Supporting Information).

To guarantee the comprehensive performance and resilience of the adhesive across diverse loading scenarios, it is essential to consider both in‐plane and out‐of‐plane strengths (Figure 4I).^[^ ^35^ ^]^ In‐plane strength, as determined through lap shear tests, evaluates the adhesive's capability to withstand forces parallel to the adhesive layer. In contrast, out‐of‐plane strength is critical for determining the resistance to perpendicular forces acting against the adhesive layer, thereby addressing concerns such as peel or delamination. The assessment of out‐of‐plane strength involved bonding stainless steel to wood and subjecting the assembly to lateral impact testing (Figure S9, Supporting Information). Notably, the specimens withstood numerous frontal impacts without failure, successfully passing the out‐of‐plane stability evaluation. Additionally, a drop test was employed to further investigate the adhesive's impact resilience and overall durability (Figure 4J; Movie S2, Supporting Information). In this experiment, a pair of wood substrates, with an adhesive area of ≈10 cm^2^, were dropped from a height of 3 meters, enduring over 20 impacts from various directions. The samples displayed no signs of cracking or displacement following impact, and the bonded region maintained its integrity consistent with the pre‐impact state. These results demonstrate that the PAA adhesive possesses substantial impact resistance and exceptional stability.

Solvent resistance is an indispensable criterion for evaluating adhesive stability; accordingly, the adhesive's compatibility with a range of organic solvents was meticulously examined. Cured specimens were immersed in ten different organic solvents: dimethyl sulfoxide, acetonitrile, chloroform, dichloromethane, 1,2‐dichloroethane, ethyl acetate, petroleum ether, toluene, ether, and silicone oil. Following a 4‐h immersion period, the adhesive strength was quantitatively evaluated (Figure 4K). For low‐polarity solvents, including petroleum ether, toluene, silicone oil, and diethyl ether, the adhesive strength exhibited minimal alteration. Conversely, with escalating solvent polarity, a decreasing trend in adhesive strength was detected. Significantly, even within the context of the most polar solvent assessed, dimethyl sulfoxide, the adhesive strength was preserved at 11.03 MPa, underscoring the adhesive's remarkable resistance to organic solvents. A slight degree of adhesive swelling at the interface was observed in the more polar solvents, which could potentially account for the observed reduction in adhesive strength.

### 2.4 Sustainability and Industrial Feasibility

**Figure 5:**
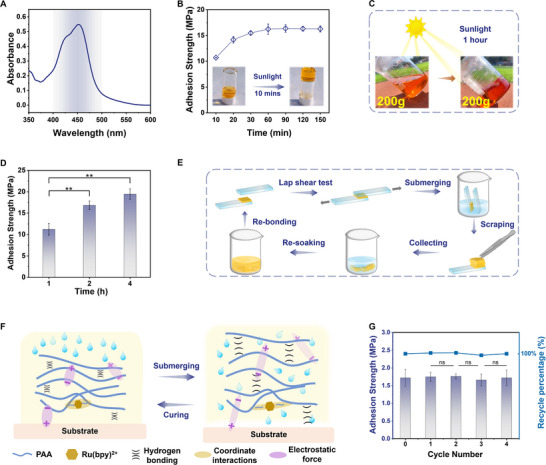
Recyclability and industrial feasibility of the PAA adhesive. A) UV–Vis spectrum of [Ru(bpy)_3_]Cl_2_ in Aqueous Solution. B) Preparation of the PAA adhesive via solar irradiation exposure. Adhesion strengths under varying exposure durations to sunlight (on wood). C) Fabrication of 200 g PAA adhesive via solar irradiation exposure. D) Adhesion strength under varying exposure durations to sunlight in large‐scale production (200 g, on wood). E) Schematic illustration of the PAA adhesive in recyclable tests. F) Schematic illustration of the assembly and disassembly process of the PAA adhesive via H_2_O volatilization and penetration. G) Adhesion strength following multiple recoveries. Data were presented as means ± standard deviation (*n* = 3). Significant levels were indicated as ^**^ *p* < 0.01.

The industrialization and deployment of novel adhesive systems demand not only the assurance of enhanced adhesion and stability but also the consideration of critical factors such as energy consumption, process complexity, and scalability for large‐scale manufacturing.^[^ ^36^ ^]^ Many high‐performance adhesives face substantial hurdles in industrialization due to these factors, which impede their widespread adoption. In the preparation of the described adhesive system, the [Ru(bpy)_3_]Cl_2_ photocatalyst, which absorbs visible light in the 400–500 nm range, was employed (**Figure** **5**A). This spectral range corresponds to ≈20% of the total solar energy. Consequently, harnessing sunlight as the light source for the initiation of the reaction offers an environmentally friendly and energy‐efficient alternative. As depicted in Figure 5B and Movie S3 (Supporting Information), the pre‐polymerized solution exhibited successful gelation following a 10‐min exposure to sunlight. The adhesion strength was found to increase progressively with extended light exposure, reaching a zenith of 16.24 MPa after 1 h, with the 30‐min adhesion strength nearly matching this maximum value (Figure 5B). The required duration of light exposure was comparable to that achieved with blue LED light. Furthermore, scaling up the process under sunlight to a larger batch size (200 g, Figure 5C) resulted in successful gelation after 1 h of light exposure, with the maximum adhesion strength of 16.84 MPa being achieved after 2 h (Figure 5D). This demonstrates the fact that the expansion of batch size does not compromise the adhesive properties.

Exceeding the adoption of renewable energy sources, the principle of reusability is instrumental in enhancing the sustainability of materials, thereby fulfilling the prerequisites for environmental compatibility. Reusability assessments were conducted on glass substrates, involving a post‐tensile testing protocol. Subsequent to the tensile test, the adhesive component was immersed in deionized water, allowing it to soften; the residual adhesive on the surface was then carefully scraped off and collected for re‐soaking and re‐bonding (Figure 5E). Upon water penetration, the hydrogen bonds and electrostatic interactions within the adhesive and at the adhesion interface are weakened, rendering the adhesive more amenable to removal from the glass substrate. During the evaporation of water, the adhesive undergoes reassemblage via hydrogen bonding and electrostatic interactions, thereby restoring its cohesive strength and enabling re‐adhesion to glass (Figure 5F). The results indicate that the adhesive strength remained consistent across successive recycling iterations (Figure 5G), substantiating the environmentally benign and recyclable characteristics of the PAA adhesive. Furthermore, the water‐solubility of the PAA adhesive contributes to the ease of substrate recycling. As illustrated in Figure S10 (Supporting Information), the stainless steel surface retained its integrity after the adhesive application and subsequent removal processes. This methodological advancement not only minimizes resource wastage but also aligns with the core principles of environmental stewardship and conservation.

### 2.5 Application

**Figure 6:**
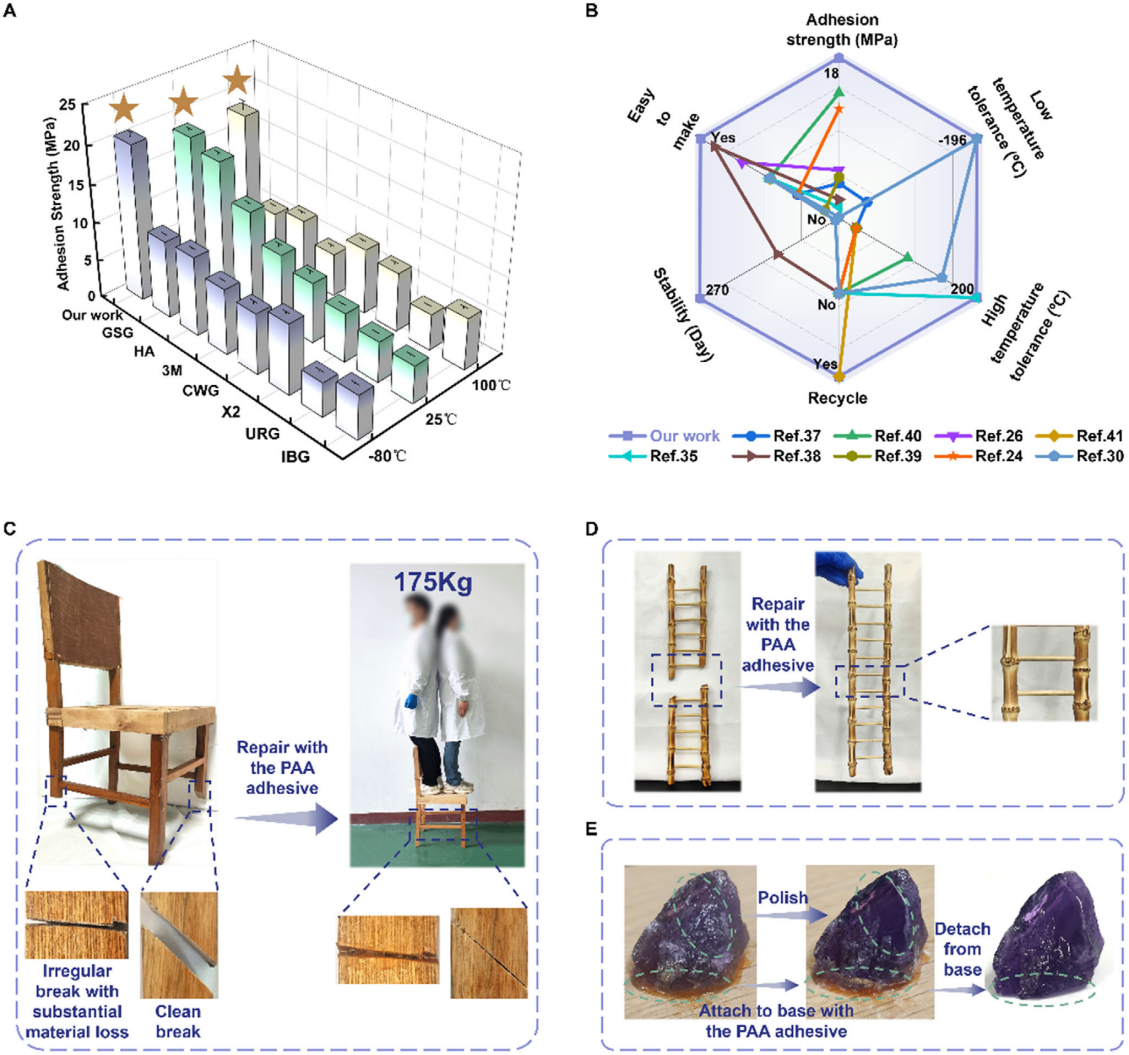
Benchmarking the PAA adhesive and its applications. A) Benchmarking of the PAA adhesive to conventional commercial adhesives on wood at −80 °C, 25 °C, and 100 °C (GSG, Gorilla Super Glue; HA, Henkel adhesive; 3M, 3M adhesive; CWG, carpentry white glue; X2, Super X2; URG, urea‐formaldehyde resin glue; IBG, industrial bone glue). Data were presented as means ± standard deviation (*n* = 3). B) Comparison of the PAA adhesive to literature‐reported water‐based adhesives and polyacrylic adhesives. C) Application of the PAA adhesive in the restoration of a chair with two fractured legs. D) Utilization of the PAA adhesive for the repair of damaged bamboo ladder handicrafts. E) Illustration of gemstone processing with the PAA adhesive.

The synthesized PAA adhesive exhibited a pronounced enhancement in adhesive performance when compared to conventional commercial adhesives specifically formulated for wood bonding applications (**Figure** **6**A). All commercial adhesives were applied and cured in accordance with their manufacturer's instructions prior to the performance evaluation. Notably, certain industrial adhesives, such as industrial bone glue (IBG), necessitate an extended period of high‐temperature pre‐dissolution, whereas others, like the urea‐formaldehyde resin glue (URG), demand the application of hot‐pressing curing, which considerably complicates the application process. At ambient temperature, the Henkel adhesive (HA, 12.65 MPa), Gorilla Super Glue (GSG, 17.10 MPa), and our PAA adhesive exhibited adhesion strengths exceeding 10 MPa. Despite the comparable adhesion strength of GSG to our PAA adhesive, it exhibited significant degradation under both low‐temperature and high‐temperature conditions, falling below 10 MPa. In stark contrast, our PAA adhesive demonstrated remarkable stability, maintaining tensile consistently near 20 MPa across a broad temperature spectrum ranging from −80 °C—100 °C. This highlights its potential as an innovative adhesive candidate. The comparative analysis of our synthesized PAA adhesive against other types of adhesives reported in the literature (such as water‐based, ionic liquid‐based, bio‐based, solvent‐free, and acrylic adhesives, Figure 6B; Tables S2 and S3, Supporting Information) underscores its superior adhesive properties, thermal resistance, recyclability, stability, and ease of preparation.^[^ ^24^, ^26^, ^27^, ^30^, ^35^, ^37^, ^38^, ^39^, ^40^, ^41^, ^42^, ^43^, ^44^, ^45^, ^46^, ^47^ ^]^ These attributes significantly broaden the scope of potential applications of PAA adhesives across various industrial sectors.

Incorporating the exceptional adhesive capabilities of the PAA adhesive, we achieved a successful restoration of a chair with two fractured legs, as depicted in Figure 6C. One leg exhibited a clean break, facilitating precise alignment, whereas the other leg featured an irregular break with substantial material loss, precluding a complete alignment. The PAA adhesive effectively restored the chair leg with a clean break and securely reattached the leg with material loss. Remarkably, following the repair, the chair was able to support weights up to 175 kg (Movie S4, Supporting Information) and withstand dynamic loads, such as stepping and jumping on its surface (Movie S5, Supporting Information), thereby emphasizing its outstanding adhesive performance. Furthermore, the versatility of the PAA adhesive is demonstrated by its application in the repair of intricate small crafts, as exemplified by the successful restoration of a bamboo ladder craft shown in Figure 6D. Its removability and robust adhesion properties make it particularly suitable for the gemstone processing industry, where it is employed to secure a variety of gemstones for precise cutting and polishing. As illustrated in Figure 6E, the adhesive can be used to secure amethyst gemstones during the carving process. Subsequent to post‐processing, the adhesive can be easily removed with water, resulting in a finely finished gemstone.

Finally, the environmental superiority of our PAA adhesive in comparison to conventional urea‐formaldehyde (UF) resin was substantiated through a series of comparative analyses (Table S4, Supporting Information).^[^ ^48^ ^]^ As a formaldehyde‐free aqueous system, PAA effectively precludes the emission of toxic formaldehyde vapors altogether, starkly differentiating from the ongoing formaldehyde release associated with UF resins. The synthesis pathways diverge fundamentally, with PAA necessitating merely ambient mixing and sunlight‐induced polymerization, in contrast to UF's reliance on energy‐intensive, alkali‐acid‐alkali catalyzed polycondensation that is sustained at a constant 90 °C. Moreover, PAA achieves curing at room temperature without the necessity of pressure application, in stark contrast to UF's requirement for 170 °C hot‐pressing. These cumulative advantages position PAA as an eco‐efficient alternative, concurrently mitigating formaldehyde pollution and diminishing energy consumption throughout both the production and application phases.

## 3 Discussion

In this study, a novel acrylic water‐based adhesive has been synthesized via a visible light‐driven catalytic process, characterized by an array of remarkable properties. These attributes include minimal energy expenditure, environmental friendliness, robust adhesion strength, exceptional stability, and recyclability. The adhesive system is designed to initiate polymerization under visible light or natural sunlight, aligning with contemporary demands for eco‐conscious protection. Its application is marked by simplicity and efficiency, obviating the need for thermal curing and requiring only gentle pressure from tweezers or equivalent implements for application, with the curing process occurring at room temperature. The PAA adhesive synthesized via this method is propelled by hydrogen bonding, electrostatic interactions, and mechanical interlocking, yielding an extraordinary level of adhesion. The adhesive exhibits bond strengths of 18.33, 12.91, and 11.03 MPa on wood, bamboo, and stainless steel substrates, respectively. Moreover, the adhesive demonstrates exceptional stability across various conditions. In terms of thermal resistance, the PAA adhesive maintains its integrity and bonding strength with negligible change within a temperature spectrum of −80 °C—100 °C. Notably, the adhesive strength slightly increases to ≈20 MPa upon cooling, and it retains an impressive bond strength of 15.84 MPa even in ultra‐low temperature conditions (−196 °C), rendering it suitable for aerospace applications. Regarding storage stability, the adhesive maintains its adhesion properties for over one month of storage. Furthermore, the adhesive retains its performance after 270 days of application, highlighting its long‐term durability. In the realm of impact resistance, a wood specimen bonded with the PAA adhesive withstands multiple drops from a height of 3 meters without sustaining damage. Moreover, a chair leg with significant material loss can be fully restored to its functional state, capable of supporting normal walking and jumping activities. The recyclability of the adhesive is achieved through osmotic and volatilization processes of water, facilitating a closed‐loop recycling process. It is particularly noteworthy that the PAA adhesive can be synthesized in bulk under sunlight, which is not only energy‐efficient but also environmentally benign throughout the preparation, application, and recycling phases, without the generation of environmentally hazardous substances. In summary, this study has presented a novel, mild visible light‐catalytic system that effectively catalyzes acrylic acid polymerization. In addition to its performance advantages, this adhesive possesses significant potential for large‐scale industrial production, offering innovative avenues for the sustainable development of water‐based adhesives.

## 4 Experimental Section

### Materials

All reagents were commercially available and used without further purification. Ascorbic acid and acrylic acid were purchased from Shanghai Aladdin Biochemical Technology Co. Ltd., China. Gallic acid and 2‐(N‐3‐Sulfopropyl‐N, N‐dimethyl ammonium) ethyl methacrylate were supplied by Shanghai Bide Pharmatech Ltd., China. Tri hexahydrate (2, 2‐bipyridine) ruthenium dichloride was purchased from Anhui Zesheng Technology Co. Ltd., China. Benzyl alcohol was purchased from Beijing Mreda Technology Co. Ltd., China. 2‐chloroquinoline was supplied by Shanghai Meryer Chemical Technology Co. Ltd., China. The blue light was purchased from Shenzhen Boxin Technology Co. Ltd., China. The device features a power output of 30W and an emission wavelength range of 450–460 nm. The dimensions of the blue light was depicted in Figure S11 (Supporting Information).

### Synthesis of 2‐Benzyloxyquinoline

To a solution of sodium hydride (336 mg, 14 mmol, 2.0 equiv.) in DMF (56 mL) was added benzyl alcohol (1.08 mL, 10.5 mmol, 1.5 equiv.) at room temperature for 30 min, then 2‐chloroquinoline (1.145 g, 7 mmol, 1.0 equiv.) was added. The reaction mixture was stirred at 80 °C for 22 h. The process of reaction was monitored by TLC until completion or no obvious progress was observed. The reaction mixture was cooled to room temperature and deionized water was added to the reaction mixture, the resulting solution was transferred to the separation funnel and extracted with ethyl acetate and water. The combined organic phase was dried with anhydrous Na_2_SO_4_, the mixture was filtered and the filtrate was concentrated under vacuum obtain a crude product. This crude product was subjected to column chromatography purification using a gradient elution of petroleum ether and a petroleum ether/ethyl acetate mixture (20:1). This purification process yielded 1.482 g of the desired compound, 2‐benzoxy‐quinoline, as a white solid, with an overall yield of 90%.^1^H NMR (400 MHz, CDCl_3_): δ 7.97 (d, *J* = 8.8 Hz, 1H), 7.87 (d, *J* = 8.4 Hz, 1H), 7.70 (dd, *J* = 8.0, 1.5 Hz, 1H), 7.65 – 7.58 (m, 1H), 7.52 (d, *J* = 6.8 Hz, 2H), 7.42 – 7.34 (m, 3H), 7.34 – 7.29 (m, 1H), 6.95 (d, *J* = 8.8 Hz, 1H), 5.55 (s, 2H).

### Preparation of PAA Adhesive

According to Table S1 (Supporting Information), ascorbic acid (2.13wt.%), [Ru(bpy)_3_]Cl_2_·6H_2_O (0.020wt.%), GA (1.60wt.%) and DMAPS (1.60wt.%) were dissolved in deionized water (61.71wt.%). Then 2‐benzoxy quinoline (0.027wt.%) was dissolved in acrylic acid (31.91wt.%). The pre‐gel precursor of PAA adhesive can be obtained by mixing the above two evenly. The pre‐gel precursor was exposed to a blue light (450–460 nm, 30 W). After irradiation for 1 h, the PAA adhesive was obtained.

### Preparation of Adhesion Samples and Lap Shear Tests

The PAA adhesive was first evenly coated on the surface of one substrate. Subsequently, another substrate was placed atop the first to form an overlap area of ≈5 mm × 5 mm. The bonded area was lightly pressed with tweezers for ≈10 s. After curing, the tensile machine was used to test the lap shear strength required for sample separation. The universal tensile testing machine selected had a transducer test force range of 500 N and a tensile speed of 50 mm/ min, and at least three samples were tested under each test condition.

### Water Loss Performance Test

The PAA adhesive was placed in a plastic petri dish and placed open in a constant temperature and humidity chamber (25 °C, 20%RH). When the test time point was reached, the test weight (m) and the initial weight (m_0_) were recorded. The mass fraction after water loss [L (%)] was calculated by the following formula

(1) $$L\left(\%\right)=\frac{m}{m_{0}}\;\times 100\%$$

### Recycling‐Reusing Methods for PAA Adhesive on Glass Surfaces

The sample was prepared with PAA adhesive and then used for tensile test after curing. After tensile testing, the adhesive portion was immersed in deionized water until it softened; the remaining adhesive on the surface was then scraped off and collected for re‐soaking and re‐bonding. This process was then repeated for subsequent cycles.

### General Characterization

^1^H NMR spectra were obtained on a Bruker Avance 400 spectrometer using CDCl_3_ as the solvent. FTIR spectra were characterized by INVENIO iS10 Fourier infrared spectrometer. The lyophilized sample was ground into a fine powder for testing. Scanning electron microscopy (SEM) was performed on a high‐resolution Regulus SU8230 scanning electron microscope. Before the test, the surfaces of the samples were deposed by gold spraying for 70 s. Thermogravimetric analysis (TGA) was carried out on a Netzsch STA 449 F5 thermogravimetric analyzer with temperatures ranging from room temperature to 800 °C at a heating rate of 10 °C min^−1^ in a nitrogen atmosphere. UV‐2550 UV–Vis spectrophotometer was used to test UV–Vis absorption spectra UV–Vis spectra of the samples were obtained on a UV‐2550 UV–Vis spectrophotometer. Rheology measurements were performed on MCR 302e. The laminator model PP15‐E with a diameter of 15 mm and a gap of 1 mm was chosen. Room temperature rheology test temperature was set at 25 °C. For the variable‐temperature rheological test, the test sample was heated at a rate of 10 °C min^−1^. The lap shear adhesion strength was conducted using a YISHITE STD500 universal testing machine.

### Statistical Analysis

Data were presented as means ± standard deviation (*n* = 3). One‐way analysis of variance (ANOVA) test was performed, followed by Tukey's test for statistical analysis. Significant levels were indicated as ns = no significant difference (*p* > 0.05), ^**^ *p* < 0.01, ^***^ *p* < 0.001, ^****^ *p* < 0.0001.

## Conflict of Interest

The authors declare that they have no competing interests.

## Author Contributions

X.F. and J.C. contributed equally to this work. X.F. was responsible for the conceptualization, methodology development, data curation, drafting of the original manuscript, revision and editing, as well as visualization. J.C. contributed to data curation, visualization, methodology, and conducted part of the investigation. Y.Z., C.X., and X.Z. were involved in the investigation. Y.L. provided supervision and secured funding for the project. J.Y. contributed to the conceptualization, project administration, and funding acquisition and participated in the review and editing of the manuscript.

## Supporting information



Supporting Information

Supplemental Movie 1

Supplemental Movie 2

Supplemental Movie 3

Supplemental Movie 4

Supplemental Movie 5

## Data Availability

The data that support the findings of this study are available in the supplementary material of this article.
