# Supplementary material for: Light‐Driven PAA Adhesive: A Green Bonding Platform Integrating High‐Performance, Environmental Resilience, and Closed‐Loop Recyclability
Source: Adv Sci (Weinh). 2025 Apr 25;12(26):2503788. doi: 10.1002/advs.202503788 (PMC12245001; doi:10.1002/advs.202503788)
Supplement: Supplementary file 1 — Supporting Information [file ADVS-12-2503788-s001.docx]

**Supporting Information**

Light-Driven PAA Adhesive: A Green Bonding Platform Integrating High-Performance, Environmental Resilience, and Closed-Loop Recyclability

Xueying Fu^a,1^, Jingtian Chen^a,1^, Yuqi Zhao^a^, Yanan Liu^a^, Chenyang Xie^a^, Xuhang Zhang^b^, Yingdan Liu^b*^and Jingyue Yang^a*^

*^a^ State Key Laboratory of Metastable Materials Science and Technology, Nano-biotechnology Key Lab of Hebei Province, Applying Chemistry Key Lab of Hebei Province, Yanshan University, Qinhuangdao 066004, China*

*^b^* *Center for Advanced Structural Materials, State Key Lab of Metastable Materials Science and Technology, and College of Materials Science and Engineering, Yanshan University, Qinhuangdao 066004, China*

*Corresponding author. Email: Jingyue Yang, [yangjingyue@ysu.edu.cn](mailto:yangjingyue@ysu.edu.cn); Yingdan Liu, [ydliu@ysu.edu.cn](mailto:ydliu@ysu.edu.cn)

**Figure S1. The synthetic procedures of 2-benzyloxyquinoline.**

**
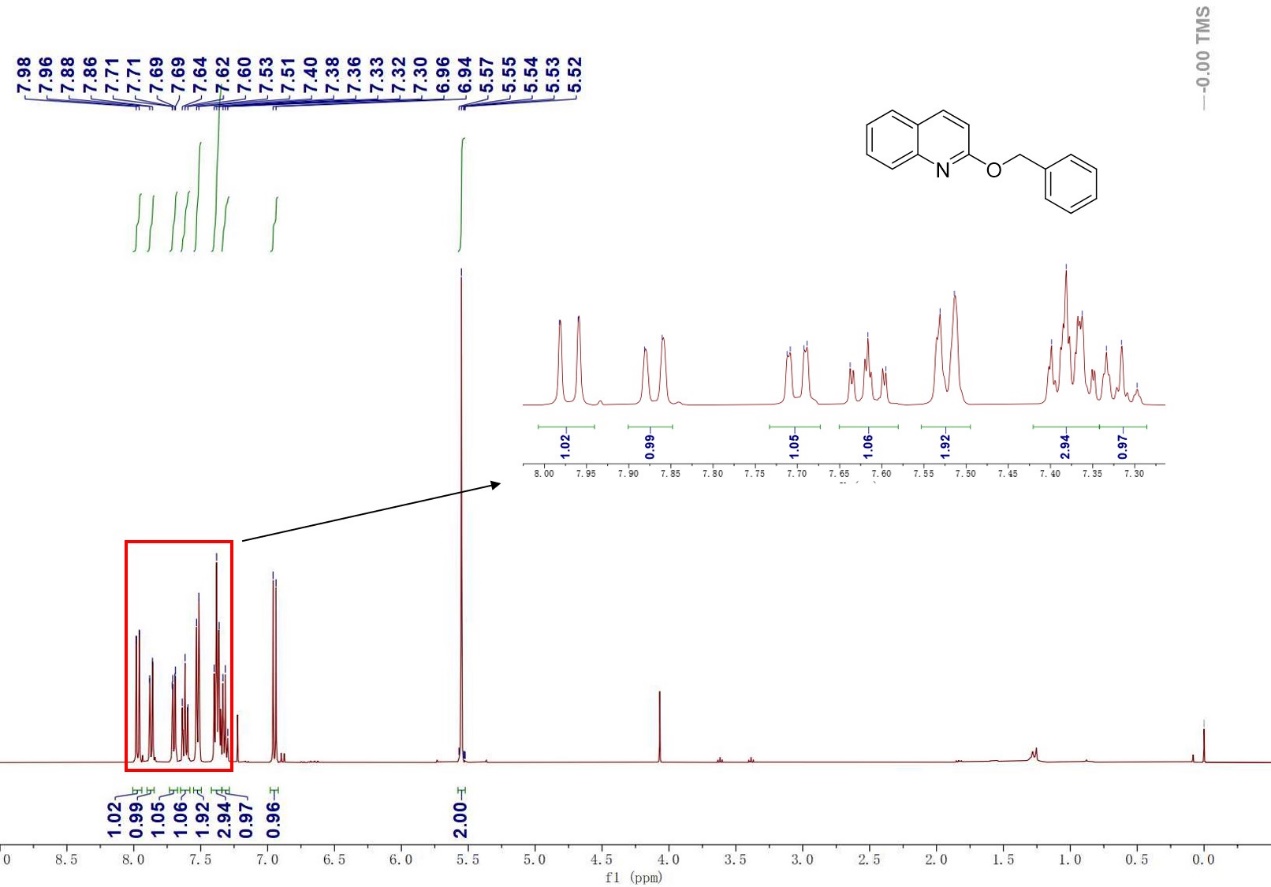
Figure S2. ^1^H NMR characterization.** ^1^H NMR spectra of 2-benzyloxyquinoline.


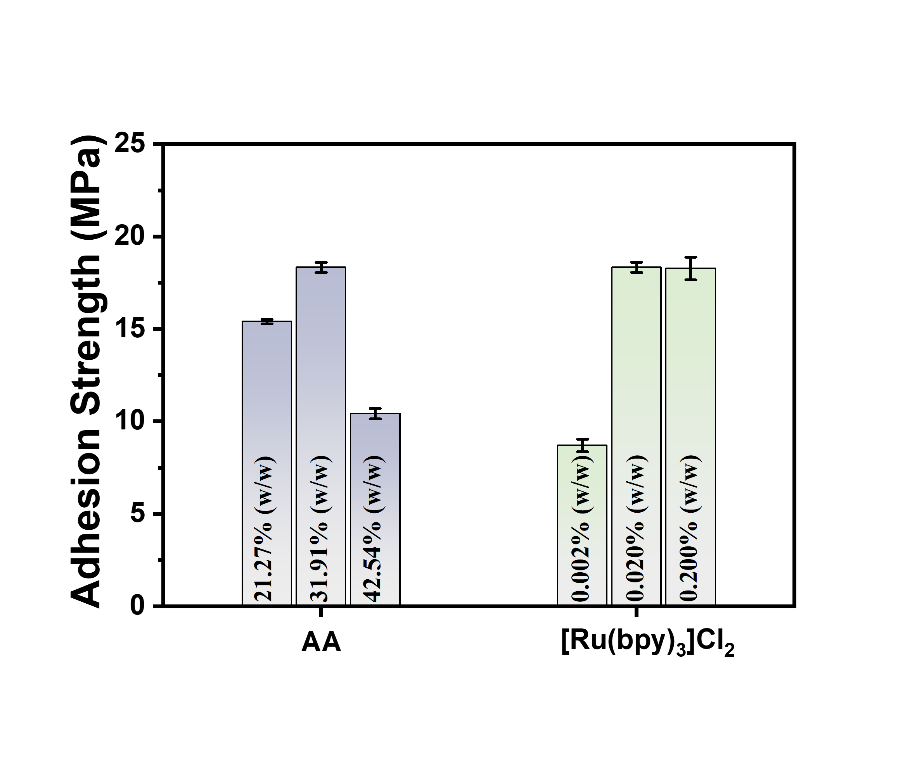


**Figure S3. Adhesion strengths of PAA adhesive with various formulations.**

**
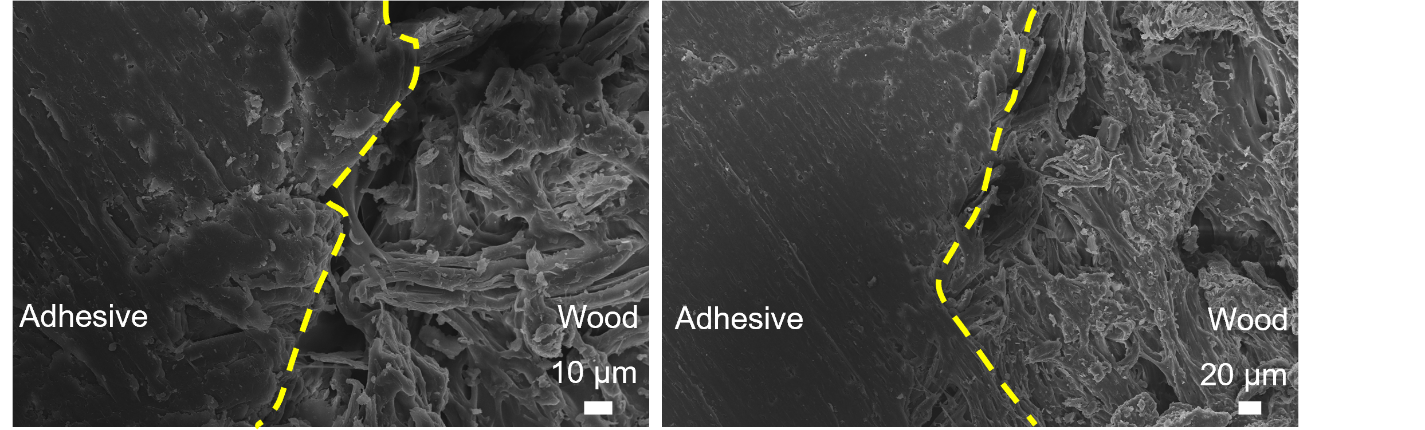
Figure S4. SEM images of the adhesive interface between PAA adhesive and wood.**

**
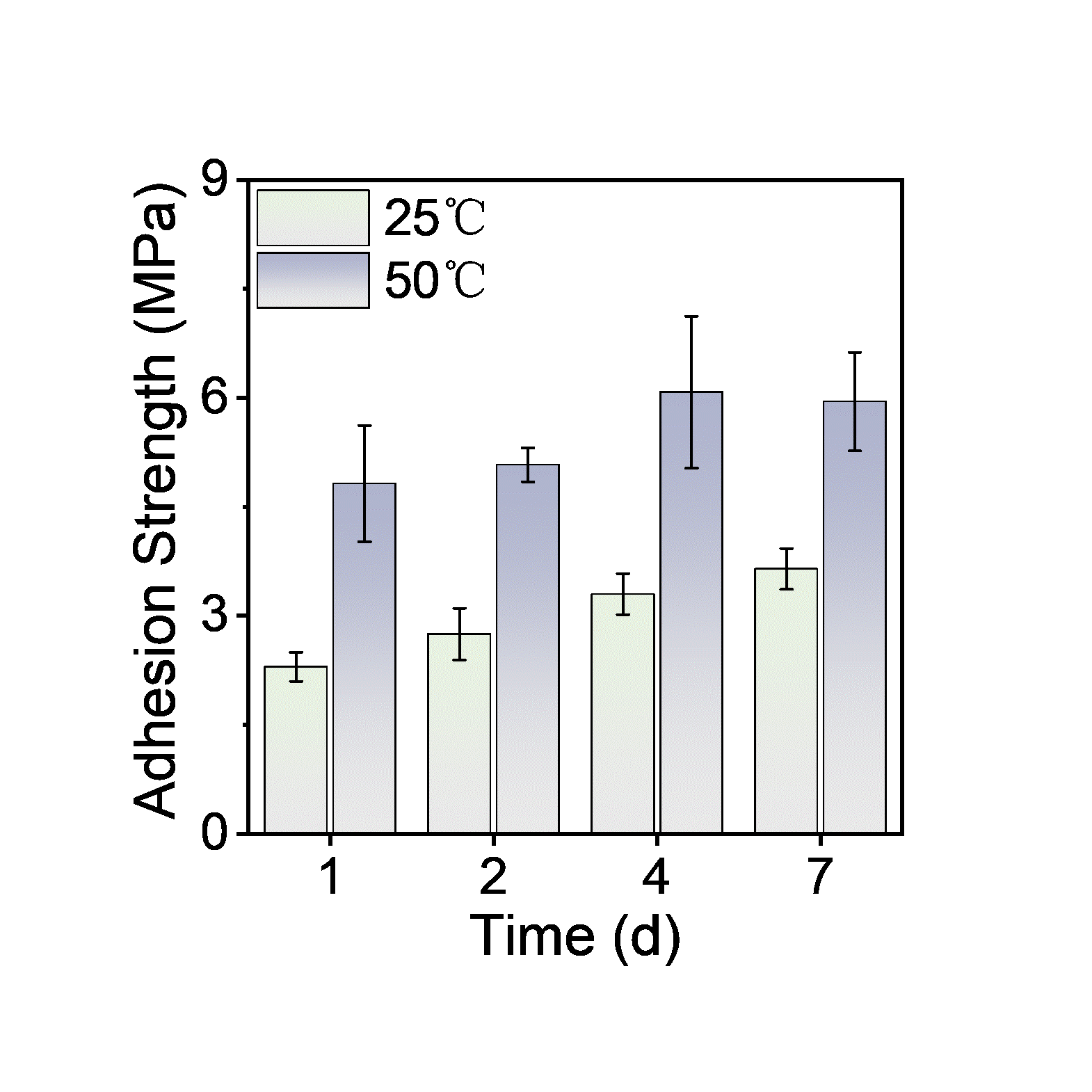
Figure S5. Adhesion strengths under different curing conditions on Ti.**

**Figure S6. Thermogravimetric analysis (TGA).** (a) TGA curve for the uncured PAA gel. (b) TGA curve of PAA adhesive cured for 24 hours at room temperature. (c) TGA curve of PAA adhesive cured for 48 hours at 50℃.

**Figure S7. Load test of PAA adhesive.** Even samples stored for over a month under (a) -20℃, (b) 25℃, and (c) 75℃ (with an adhesive area of approximately 12 cm²) still supported a 25 kg load without any fractures or displacement.


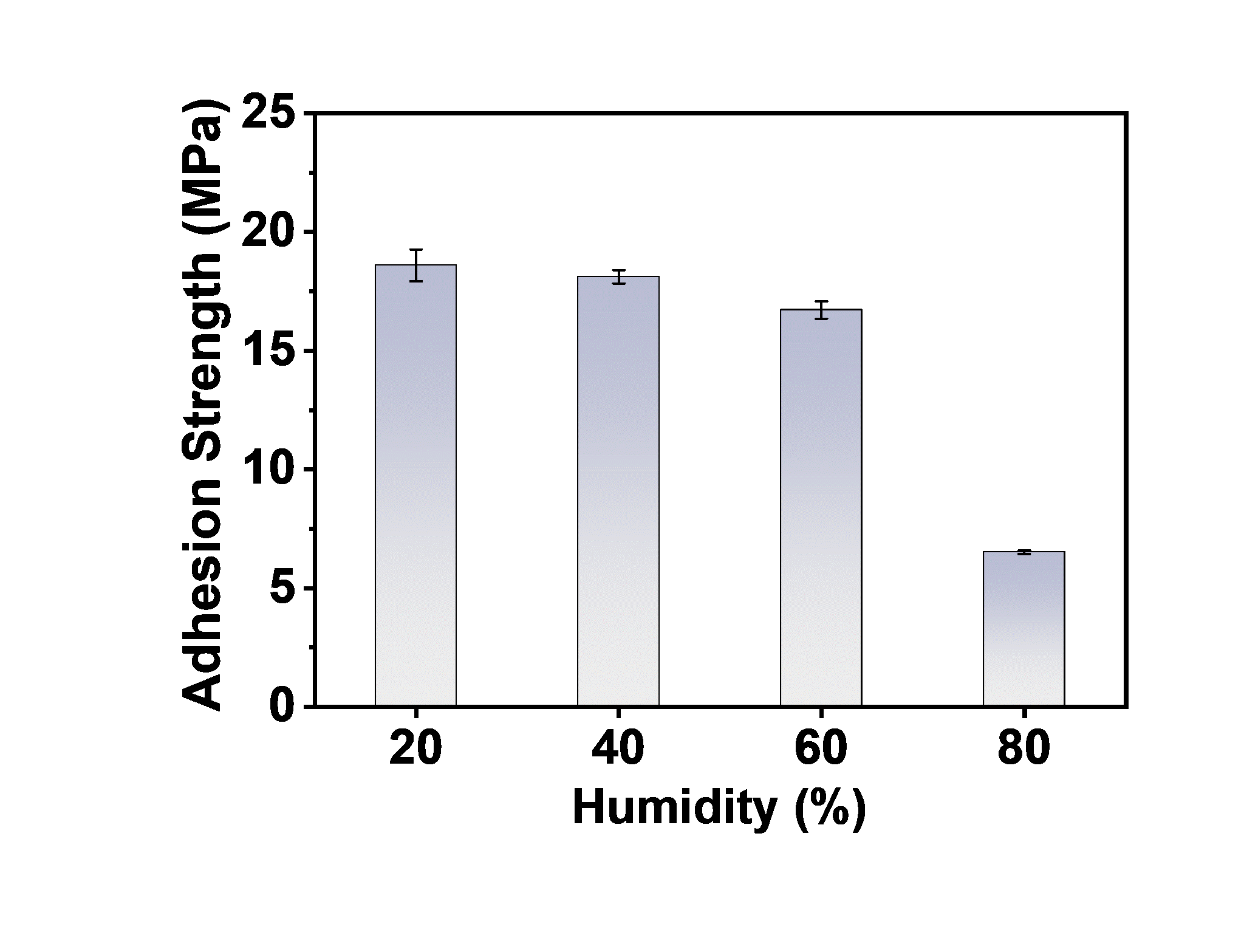
**Figure S8. Adhesion strengths at various humidity.** Lap shear adhesion strength of PAA adhesive in various humidity environments for 12 hours.

**Figure S9. The assessment of out-of-plane strength.** Bonding stainless steel to wood and subjecting the assembly to lateral impact testing.

**Figure S10. SEM images of stainless steel surface before (left) and post (right)-adhesive application.**

**
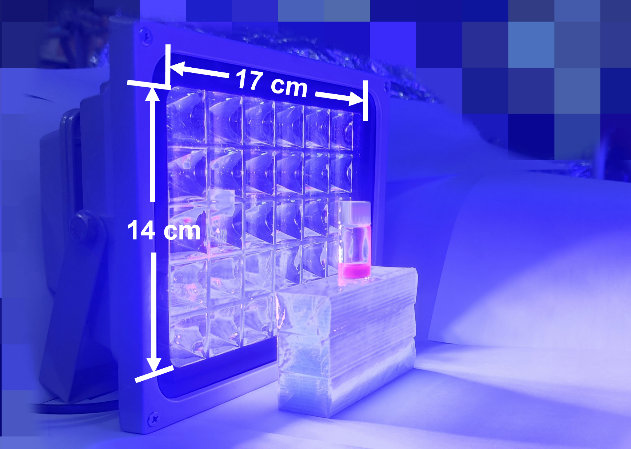
**

**Figure S11. Graphical illustration of the blue light employed in the photocatalytic process.**

**Table S1. The formulation of PAA adhesive**

| Ingredients | 2-benzyloxyquinoline | Ascorbic acid | [Ru(bpy)_3_]Cl_2_·6H_2_O | Acrylic acid | GA | DMAPS | H_2_O |
| --- | --- | --- | --- | --- | --- | --- | --- |
| Concentrations  [wt%] | 0.027 | 2.13 | 0.020 | 31.91 | 1.60 | 1.60 | 62.71 |

**Table S2. Comparison of PAA adhesive with other reported water-based adhesives and polyacrylic adhesives in adhesion strength, low temperature resistance, high temperature resistance, recyclability and stability**

| Samples | Substrates | Adhesion strength (MPa) | Low temperature tolerance (℃) | High temperature tolerance (℃) | Recyclable | Stability (Day) | | Refs |
| --- | --- | --- | --- | --- | --- | --- | --- | --- |
| **Our work** | **Wood**  **Bamboo**  **Steel** | **18.33**  **12.91**  **11.03** | **-196 (15.84 MPa)** | **200 (6.42 MPa)** | **Yes** | **> 270** | **-** | |
| SP/LPU | Wood  Steel | 4.00  2.70 | -40 (2.17 MPa) | RT | No | - | *(37)* | |
| PVA/PAA | Wood | 14.30 | - | 100 (5.7 MPa) | No | - | *(40)* | |
| PA/b-PEI | Wood  Steel | 5.52  1.70 | - | RT | No | - | *(26)* | |
| P@TS/AJ | - | - | - | RT | Yes | - | *(41)* | |
| ChNC | Wood  Glass | 1.30  1.74 | - | 200 (1.19 MPa) | No | - | *(35)* | |
| WPU/PCD | Zn  Al  Fe | 2.15  2.00  2.10 | - | RT | No | 120 | *(38)* | |
| SF-PDA | the glass slides  steel  Al | 2  4.7  2.2 | - | RT | No | - | *(39)* | |
| PAA-DES | glass  Steel/glass | 12.50  8.63 | - | RT | No | - | *(24)* | |
| PTA | glass | 0.13 | -196 (NA) | 150 (NA) | No | 7 | *(30)* | |

**Table S3. Comparison of PAA adhesive with** **other types of adhesives (such as** **solvent-free adhesives,** **ionic liquid-based adhesives or** **bio-based adhesives) in adhesion strength, low temperature resistance, high temperature resistance, recyclability and stability**

| Samples | Substrates | Adhesion strength (MPa) | Low temperature tolerance (℃) | High temperature tolerance (℃) | Recyclable | Stability | | Refs |
| --- | --- | --- | --- | --- | --- | --- | --- | --- |
| **Our work** | **Wood**  **Bamboo**  **Steel** | **18.33**  **12.91**  **11.03** | **-196 (15.84 MPa)** | **200 (6.42 MPa)** | **Yes** | **> 270 Days** | **-** | |
| CHITA (bio-based adhesive) | Al/Steel | 3.9 | - | RT | No | 73 Days | *(43)* | |
| BSA (bio-based adhesive) | Steel | 14.6 | -196 (9.5 MPa) | RT | No | - | *(44)* | |
| NRL/PVA (bio-based adhesive) | Cardboard  Wood | 1.31  1.65 | -18 (0.9 MPa) | 100 (1.47 MPa) | No | 14 Days | *(42)* | |
| Tri-HT (ionic liquid-based adhesive) | Wood  Glass  Ceramic | 2.79  4.77  10.50 | -20 (5 MPa) | 55 (0.3 MPa) | No | 30 Days | *(27)* | |
| p(Elp-TA) + PVP (solvent-free adhesive) | Cu | 1.12 | - | RT | No | - | *(47)* | |
| PUIP-NAGA (solvent-free adhesive) | Steel | 7.57 | -50 (3.1 MPa) | RT | No | 30 Days | *(46)* | |
| LTFe (solvent-free adhesive) | Steel | 0.02 | -20 (0.1 MPa) | 37 (0.32 MPa) | No | 56 Days | *(45)* | |

**Table S4.** **The environmental superiority of PAA adhesive in comparison to conventional urea-formaldehyde (UF) resin**

| sample | PAA adhesive (this work) | urea–formaldehyde (UF) resin^[48]^ |
| --- | --- | --- |
| preparation process | sunlight exposure after mixing | alkali-acid-alkali method and two time polycondensations |
| preparation temperature | ambient temperature | continuous high temperature (90℃) |
| curing method | setting at ambient temperature | hot-pressing (170℃) |
| main energy source | solar power | electric energy |
| formaldehyde emission (mg/100 g) | 0 | 2.56 |
